# Optimization of a polyphenol extraction method for sweet orange pulp (*Citrus sinensis L*.) to identify phenolic compounds consumed from sweet oranges

**DOI:** 10.1371/journal.pone.0211267

**Published:** 2019-01-30

**Authors:** Lisard Iglesias-Carres, Anna Mas-Capdevila, Francisca I. Bravo, Gerard Aragonès, Begoña Muguerza, Anna Arola-Arnal

**Affiliations:** 1 Universitat Rovira i Virgili, Departament de Bioquímica i Biotecnologia, Nutrigenomics Research Group, Tarragona, Spain; 2 Eurecat, Centre Tecnològic de Catalunya, Technological Unit of Nutrition and Health, Reus, Spain; Tallinn University of Technology, ESTONIA

## Abstract

The consumption of sweet oranges has been linked to several health benefits, many of which are attributed to hesperidin, a flavanone that is present in high amounts in these fruits. However, other phenolic compounds can contribute to the bioactivity of sweet orange. To link those effects to their phenolic profile, the complete characterization of the phenolic profile is mandatory. Although many studies have profiled the phenolic composition of orange juices, their pulps, which retain phenolic compounds, are overlooked. This fact is particularly relevant because dietary guidelines recommend the consumption of whole fruits. Therefore, this study aimed to develop a specific method for the optimal extraction of phenolics from orange pulp and to use this method to characterize these fruits grown at different locations by HPLC-ESI-MS/MS. The extraction conditions that reported the highest total polyphenol content (TPC) and hesperidin contents were 20 mL/g, 55 °C, and 90% methanol. The extraction time and number of sequential steps were further evaluated and optimized as 20 min and two extraction steps, respectively. Although lower extraction rates were achieved when using ethanol as the extraction solvent, high TPC and hesperidin yields were obtained, suggesting the potential use of this methodology to produce phenolic-rich extracts for the food industry. By applying the optimized methodology and analyzing the extracts by HPLC-ESI-MS/MS, geographic cultivation regions were demonstrated to affect the phenolic profiles of oranges. In short, we developed a quick, easy-to-perform methodology that can be used to extract orange phenolics from pulp for their identification and quantification and to evaluate the factors that affect the phenolic profile in sweet orange pulps.

## Introduction

Phenolic compounds are secondary plant metabolites that can enter the human diet though the consumption of vegetal products. Importantly, a wide range of health-related activities have been reported for phenolic compounds. In this sense, the phenolic compounds present in citrus fruits have received attention due to their anti-oxidant, anti-inflammatory and cardioprotective activities [1]. Sweet oranges are a rich source of flavanones, and the effects of their consumption are related to cardioprotective effects, among others [2,3]. Remarkably, hesperidin, the most abundant flavanone in sweet oranges [1,4,5], is believed to be the responsible for many of the biological effects linked to sweet orange consumption [1–3]. Other relevant compounds in sweet oranges that can contribute to their health-promoting activities include narirutin, phenolic acids and flavonols [4,6,7].

To link the consumption of sweet orange phenolic compounds to a health effect, characterization of their phenolic profile is required. For flavanones specifically, their extraction and analysis remain challenging due to their chemical complexity and vast distribution in the plant kingdom [1]. Additionally, factors such as the solvent type, temperature, extraction time and liquid-solid ratio (LSR) influence the polyphenol extraction yield [8–11]. Consequently, it becomes difficult to develop a universal extraction method for all the phenolic compounds in all food matrixes [8]. Hence, the extraction method must be optimized for each food matrix. In this sense, response surface methodology (RSM) has been used to optimize the extraction of polyphenols in several plant products, including fruits [9,11–19]. This methodology allows the evaluation of interactions between different independent variables and their effects on the response variables [9].

There are multiple optimized methods for extracting phenolics from citrus peels [10,17,19–21], and many studies have analyzed orange juice without any extraction methods [6,22,23]. Indeed, the phenolic content of orange juices has been widely profiled [6,7,22,23], and the factors that modulate it, which include orange variety, maturity stage, post-harvest processing and storage conditions, have been studied [5,24,25]. However, orange pulps, which are the edible parts of sweet oranges, are usually overlooked. Although some attempts have been performed to characterize the whole edible parts of oranges, no specific or optimized extraction methodologies have been used. Additionally, only flavanone aglycones and total phenolics were quantified, which is insufficient to link the consumption of a fruit to a specific health benefit [26–28]. Importantly, orange pulp by-products are known to contain phenolic compounds [29,30]. Therefore, only profiling citrus juices can lead to a lower quantification of the real phenolic content in this type of fruit. Additionally, the phenolic profiles of citrus peels, pulps and juices differ. More specifically, the phenolic profile of citrus peels differs from citrus pulps and juices in terms of the type of phenolic compounds, but the differences in the phenolic profiles of citrus juices and pulps are mainly quantitative [3,4,31]. Further, dietary guidelines recommend the consumption of whole fruits instead of juices [32,33]; in the specific case of sweet oranges, this includes their pulps.

Given the chemical diversity of phenolic compounds [1], the different food matrixes [5,34] and the influence of the extraction variables [8–10], specific extraction methods must be developed. However, to our knowledge, there is no optimized extraction method for isolating orange pulp phenolics. Therefore, the aim of this study was to determine the best extraction conditions for polyphenols from sweet orange pulp by RSM. Additionally, the phenolic profile of two different Navelina sweet oranges cultivars was characterized by HPLC-ESI-MS/MS to evaluate whether the geographical growing location could modulate the phenolic profiles of sweet oranges.

## Materials and methods

### Plant material

Navelina oranges (*Citrus sinensis L*.), grown in the southern (SO) or northern (NO) hemisphere, were purchased form Mercabarna (Barcelona, Spain). Fresh oranges were peeled, and the pulp was frozen in liquid nitrogen. Oranges were ground until reaching homogeneity, and the homogenates were lyophilized for one week in a Telstar LyoQuest lyophilizer at—55 °C, after which the samples were further ground to obtain a fine powder. Orange powder was kept dry and protected from light exposure until extraction. NOs were used to develop the extraction method, and NOs and SOs were used to validate the extraction method and provide insights into the factors affecting the phenolic profile in oranges.

### Chemicals and reagents

Acetonitrile (HPLC analytical grade) and glacial acetic acid were purchased from Panreac (Barcelona, Spain). Methanol and ethanol (HPLC analytical grade) were purchased from Sigma-Aldrich (Madrid, Spain). Formic acid was purchased from Scharlab (Barcelona, Spain). Ultrapure water was obtained from a Milli-Q Advantage A10 system (Madrid, Spain). The Folin-Ciocalteu reagent was purchased from Fluka/Sigma-Aldrich (Madrid, Spain). Apigenin, chlorogenic acid, diosmetin, eriodictyol, eriodyctiol-7-*O*-glucoside, quercetin-3-*O*-galactoside (hyperoside), isorhamnetin-3-*O*-glucoside, kaempferol, kaempferol-3-*O*-glucoside, and kaempferol-3-*O*-rutinoside were purchased from Exrtasynthese (Genay, France). Benzoic acid, caffeic acid, p-coumaric acid, ferulic acid, gallic acid, naringenin, phloroglucinol, protocatechuic acid and quercetin were purchased from Fluka/Sigma-Aldrich (Madrid, Spain). Hesperidin was purchased from Quimigranel (Barcelona, Spain) and hesperetin, naringin and rutin were kindly provided by Nutrafur (Murcia, Spain).

Standard compounds were individually dissolved in methanol at 2000 mg/L, with the exception of naringenin and isorhamnetin-3-*O*-glucoside, which were dissolved at 1000 mg/L, and hesperidin and hyperoside, which were dissolved at 500 mg/L. All standard stock solutions were newly prepared every 3 months and stored in amber-glass flasks at -20 °C. Mixed standard stock solutions were diluted with Milli-Q water to obtain the desired concentration needed to construct the calibration curves.

### Extraction procedure

NO powder was weighed to obtain the desired LSR and mixed with 1 mL of pre-heated extraction solvent. Different methanol proportions (methanol:water, v:v), temperatures, times and extraction steps were used throughout the experiment. Additionally, in all cases, methanol was prepared with 1% formic acid. All extractions were performed with 500 rpm agitation under protection from light exposure. Samples were centrifuged at 8,500 rpm for 10 min at 4 °C, and the supernatant fractions were collected and stored at -20 °C until analysis.

### Analyses of the response variables

#### Determination of the total phenolic content

The TPC of extracts was determined by the Folin-Ciocalteu method [35]. Briefly, 50 μL of the extract and 50 μL of the Folin-Ciocalteu reagent were consecutively added to an Eppendorf tube containing 500 μL of water. After 3 min, 100 μL of Na_2_CO_3_ (25%) was added to the mixture. A final volume of 1 mL was reached by the addition of Milli-Q water. The absorbance was read at 725 nm using an Eon BioTek spectrophotometer (Izasa, Barcelona, Spain) against a water sample that underwent equal treatment after 1 hour of incubation in the dark. Gallic acid at different concentrations was used as a standard compound to construct the calibration curves. The results were expressed as milligram gallic acid equivalents per gram of dry weight (GAE/g dw).

#### HPLC-DAD analysis of phenolic compounds

An HPLC-DAD method was developed to quantify hesperidin in NO powder. Polyphenol separation was achieved using a ZORBAX Eclipse XDB-C18 (150 mm x 2.1 mm i.d., 5 μm particle size) as the chromatographic column (Agilent Technologies, Palo Alto, CA, USA) equipped with a Narrow-Bore guard column (2.1 mm x 12.5 mm 5 μm particle size, Agilent Technologies, Palo Alto, CA, USA). The mobile phase was 0.25% acetic acid in water (A) and acetonitrile (B) in a gradient mode as follows: initial conditions, 0% B; 0–1 min, 0–20% B; 1–8 min, 20–30% B; 8–9 min, 30–100% B; 9–10 min, isocratic at 100% B; and 10–11 min, 100–0% B. A 5-min post-run was applied for column equilibration. The flow rate was set at 0.5 mL/min, and the injection volume was 10 μL for all runs.

Identification and quantification of hesperidin was achieved with a UV/VIS photodiode array detector (1260 Infinity, Agilent Technologies, Palo Alto, CA, USA). Chromatograms were recorded from 200 to 400 nm. Hesperidin was detected and quantified at 280 nm [7]. The results were expressed as mg hesperidin/g dw and were used in the RSM design to evaluate the effect of multiple extractions and solvent replacement on the extraction of orange phenolic compounds.

To validate the quantitative HPLC-DAD method, the calibration curves, linearity, intraday variability (precision), interday variability (reproducibility), and detection and quantification limits were calculated using Milli-Q water spiked with hesperidin. The peak areas of the various hesperidin concentrations were used to construct calibration curves. The method’s precision was determined as the relative standard deviation (% RSD) of the concentration in a triplicate analysis from three different spiked samples (10, 200 and 500 μg/mL). Method reproducibility was determined as the relative standard deviation (% RSD) of the concentrations from three different hesperidin concentrations (10, 200 and 500 μg/mL) analyzed in triplicate over three consecutive days. Sensitivity was evaluated by determining the limits of detection (LOD) and quantification (LOQ), which are defined as the concentration corresponding to 3-fold and 10-fold of the signal-to-noise ratio, respectively. The quality parameters are summarized in S1 Table.

#### HPLC-ESI-MS/MS

The extracts were directly analyzed using a 1200 LC Series coupled to a 6410 MS/MS (Agilent Technologies, Palo Alto, CA, USA). The column and mobile phases used were the same as in the HPLC-DAD method (see section 2.4.2). The gradient mode was used with the following conditions: initial condition, 0% B; 0–0.5 min, 0% B; 0.5–2 min, 0–10% B; 2–12 min, 10–30% B; 12–16 min, 30–60% B; 16–17 min, 60–100% B, 17–20 min, 100% B; and 20–21 min, 100–0% B. A 6-min post-run was required for column re-equilibration. The flow rate was set at 0.4 mL/min, and the injection volume was 2.5 μL for all runs. Electrospray ionization (ESI) was conducted at 200 °C and 14 L/min with 20 psi of nebulizer gas pressure and a capillary voltage of 3000 V. The mass spectrometer was operated in the negative mode and the MS/MS data were acquired in dynamic mode. The optimized conditions for the analysis of the phenolic compounds studied using HPLC-ESI-MS/MS are summarized in S2 Table. Data acquisition was performed using the MassHunter Software (Agilent Technologies, Palo Alto, CA, USA). The calibration curves, coefficient of determination, linearity and detection and quantification limits for the HPLC-ESI-MS/MS method can be found in S3 Table and were evaluated following the same principles as in the HPLC-DAD method (see section 2.4.2). The Total Ion Chromatogram (TIC), individual MRM responses for the quantified compounds in NO and SO and their spectra can be found in S1–S3 Figs.

### Experimental design

#### Range selection

Prior to RSM, the extraction temperature, methanol concentration and LSR effects were individually evaluated on the extraction of the TPC from NOs to select the RSM working ranges. The temperature was evaluated at 25, 40, 55, 70 and 85 °C; methanol at 30, 50, 60, 70, and 90%; and LSR at 5, 10, 20, 30 and 40 mL/g. When not evaluated, the extraction parameters were kept constant at 55 °C, 50% and 20 mL/g, respectively. The extractions at this stage of the optimization were performed for 30 min (500 rpm).

#### Response surface methodology

The extraction was optimized using an experimental design by RSM. A face-centered central composite design with two factors and three levels consisting of 11 randomized runs with 3 center-point replicates was selected. The independent variables were the extraction temperature (25–55 °C; X1) and methanol concentration (50–90% methanol:water, 1% formic acid, X2). LSR and the extraction time were fixed at 20 mL/g and 30 min, respectively. The experimental data were fit to a second polynomial response surface using the equation

$$Y=\beta_{0}+\sum_{i=1}^{2}\beta_{i}X_{i}+\sum_{i=1}^{2}\beta_{ii}X^{2}{}_{ii}+\sum_{i=1}\sum_{j=i+1}\beta_{ij}X_{ii}X_{ji}$$ (1)

where Y is the dependent variable, β0 the constant coefficient, and βi, βii and βij are the linear, quadratic and interaction regression coefficients, respectively. Xi, Xii and Xji represent the independent variables.

TPC and hesperidin levels were used as response (dependent) variables in the RSM optimization step. The results from the RSM design were analyzed with the Design-expert 9.0.6 software (Trial version, Stat-Ease Inc., Minneapolis, MN, USA).

#### Extraction time

The contribution of the extraction time to the NO TPC was evaluated by performing phenolic extractions at 55 °C using 90% methanol (1% formic acid) with an LSR of 20 mL/g within a range from 0 to 120 min (500 rpm). An extraction time of 0 min was established for samples that were mixed with the extraction solvent and immediately centrifuged (8,500 rpm for 10 min at 4 °C).

#### Multi-step extractions

To evaluate the effect of multiple extraction steps on the recovery of the TPC and hesperidin from NOs, three sequential extractions were performed on orange powder under the following conditions: 55 °C, 90% methanol (1% formic acid), and 20 min (20 mL/g, 500 rpm).

#### Ethanol-methanol comparison

To evaluate the efficiency of ethanol on TPC and hesperidin extraction, NO powder was extracted twice under the following conditions: 55 °C, 90% methanol or ethanol (1% formic acid), and 20 min (20 mL/g, 500 rpm).

### Statistical analysis

The experimental results for the RSM design were analyzed using the Design-Expert 9 software (Trial version, Stat-Ease INC., Minneapolis, MN, USA). The SPSS 19 software (SPSS Inc., Chicago, IL, USA) was used for any other statistical analysis. All experiments were performed in triplicate and the statistical significance was evaluated using a one-way ANOVA or Student’s t-test. A p-value less than 0.05 was considered to be statistically significant.

## Results and discussion

Citrus polyphenols such as hesperidin are known to exert beneficial health effects when consumed [1–3]. The proper quantification of a food matrix is essential to correlate its consumption and an observed health effect. The diversity of phenolic compounds found among fruits [1], fruit varieties [5] and even fruit parts [31] makes it difficult to develop a universal extraction method. Therefore, specific extraction methods must be developed to accurately profile the phenolic composition of a plant matrix. Moreover, no optimized extraction methods have been developed for orange pulps: they not only contain substantial quantities of phenolic compounds [29,30], but their consumption is also recommended over that of their juices [32,33]. To determine the optimal extraction conditions for orange pulp, RSM was applied to optimize the TPC and as the main compound occurring in sweet oranges, hesperidin [5]. Throughout the optimization process, NOs were used due to their higher phenolic contents.

### Range selection

**Fig 1:**
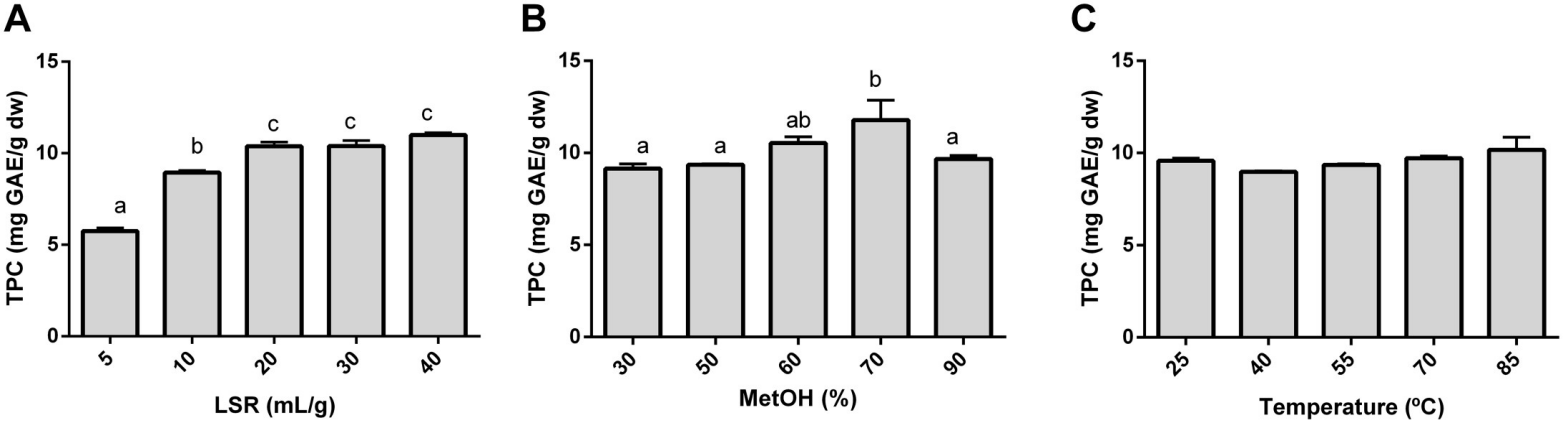
Individual effect of LSR (A), methanol proportion (B) and temperature (C) on the extraction of total phenolic compounds from *Citrus sinensis* pulp. The results are expressed as mg of gallic acid equivalents per g of dry weigh ± SD (mg GAE/g dw). ^a, b, c^ Mean values (n = 3, each) with different letters denote significant differences by one-way ANOVA with Tukey’s post-hoc test (p<0.05).

Prior to RSM, the LSR, temperature and methanol proportion effects were individually evaluated for the extraction of the TPC from NOs (Fig 1). This allowed the selection of an optimized LSR and the selection of RSM working ranges for methanol and temperature. The extraction time was fixed at 30 min according to other studies [9,12].

The selection of an appropriate LSR is of significant relevance when optimizing an extraction method, as low LSRs lead to solvent saturation and high LSRs are economically inefficient [36]. Therefore, the LSR was evaluated at 5, 10, 20, 30 and 40 mL/g. As the solvent proportion increased, the TPC extraction increased, up to 20 mL/g (Fig 1A). In this sense, Inoue *et al*. reported changes in the hesperidin and narirutin extraction yields due to LSR variations in kiyomi (*Citrus unshiu*) peels [10], which are the most abundant phenolics in oranges. However, no significant differences were observed at values higher than 20 mL/g. Therefore, the LSR was fixed at 20 mL/g for the rest of the experiments. Similar LSR values were also reported to be optimal for the extraction of phenolics from sun-dried apricots [12] and dried mandarin (*Citrus reticulate*) peels [20]. Additionally, Silva *et al*. reported a similar behavior for TPC extraction from *Inga edulis* leaves [9] and Pompeu *et al*. from *Euterpe oleracea* fruits [13].

Throughout these experiments, methanol was always prepared with formic acid at a concentration of 1%. Low concentrations of organic acids enhance the extraction of phenolics by promoting plant matrix degradation [15]. The solvent type and percentage can greatly influence the extraction of phenolics in citrus species [37,38] and other plant materials [14,15]. The effect of the methanol proportion (Fig 1B) observed in this study is consistent with the fact that phenolic extractability increases with increasing solvent proportions to a certain point, after which it starts to decrease [11,16], as reported for other citrus species [10,17]. The maximum phenolic extraction was reached at 70% methanol. Therefore, 50, 70 and 90% methanol were chosen as the low, medium and high methanol percentages, respectively, for the RSM.

Temperature usually affects the extraction of phenolics by enhancing the solubility of phenolic compounds [39]. However, an increase beyond a certain point can promote the degradation of phenolics due to thermal and enzymatic degradation [40]. Nevertheless, no significant effects of temperature were observed for the extraction of the TPC from oranges within the range studied (Fig 1C); this has also been previously described by S. Chang *et al*. in the extraction of the TPC from kaffir lime (*Citrus hystrix*) peels [17]. Therefore, relatively mild temperatures of 25, 40 and 55 °C were selected to be applied as the low, medium and high levels, respectively, in the RSM. Additionally, working at mild temperatures is recommended in such studies as high temperatures promote extraction solvent loss due to vaporization, which increases the extraction cost from the food industry’s point of view [17].

### Response surface methodology

The phenolic extraction from orange pulp was further optimized by RSM. Throughout the experimental RSM step optimization, the LSR was fixed at 20 mL/g, as previously determined, and the extraction time was set at 30 min according to other studies [9,12]. A face-centered central composite design with two factors and three levels consisting of 11 randomized runs with 3 center points was selected. The independent variables used in the RSM were temperature (25–55 °C, X_i_) and the methanol proportion (50–90%, X_j_). TPC and hesperidin contents were included in the model. Selection of these variables was based on the evaluation of all the phenolic compounds present in sweet orange pulps based on the TPC and the importance of hesperidin in sweet oranges as the main phenolic compound [5] for health promoting activities [1–3].

#### Multiple linear regression and model adequacy

The TPC and hesperidin results from all the runs are shown to be dependent on the extraction variables in Table 1. These experimental data were used to determine the regression coefficients in Eq 1. While hesperidin extraction fit a quadratic model, TPC fit a linear one (Table 2). Good fit was achieved and the responses’ variability was explained by the model. Additionally, the determination coefficients (R^2^) for TPC and hesperidin were higher than 0.9. ANOVA analysis was statistically significant, suggesting that at least one of the model parameters could explain the variation in the responses in relation to the average response. Furthermore, statistical insignificance (*p* > 0.05) in the lack of fit test showed that the model properly fit the experimental data, thus further validating the model.

**Table 1:** Face-centered settings for the independent variables and experimental results for hesperidin and the total phenolic content (TPC) in *Citrus sinensis* pulp.

Run	T^a^ (°C)	MetOH (%)	Hesperidin (mg/g dw)	TPC (mg GAE/g dw)
1	25	50	8.60	8.85
2	55	50	11.08	9.59
3	25	90	18.20	10.75
4	55	90	20.17	11.55
5	25	70	16.68	10.37
6	55	70	18.14	10.46
7	40	50	8.19	9.35
8	40	90	18.86	10.78
9	40	70	16.07	9.93
10	40	70	15.86	10.30
11	40	70	16.13	10.18

Abbreviations: T^a^, temperature; MetOH, methanol; and GAE, gallic acid equivalents.

**Table 2:** Analysis of the variance and regression coefficients for the predicted model for the response variables in Citrus sinensis pulp.

Model parameters	Regression coefficients	Hesperidin	TPC
Intercept	β_0_	-29.62	6.38
Lineal			
T^a^	β_1_	-0.32**	1.82x10^-2^*
MetOH	β_2_	1.21**	4.41x10^-2^**
Interaction	β_12_	-4.25x10^-4^	-
Quadratic			
T^a^xT^a^	β_11_	5.24x10^-3^**	-
MetOH x MetOH	β_22_	-6.77x10^-3^**	-
p-value		<0.0001	<0.0001
R^2^		0.9946	0.9191
Adjusted R^2^		0.9892	0.8989
F-value		184.3	45.44
Lack of fit ^a^		0.0636	0.4094

Abbreviations: T^a^, Temperature; MetOH, methanol; and R^2^, determination coefficient.

Differences between the groups were determined by ANOVA,

* *p* < 0.05,

** *p* < 0.01.

^a^ p-value of the lack of fit test.

#### Analysis of the regression coefficients and response surface plots

**Fig 2:**
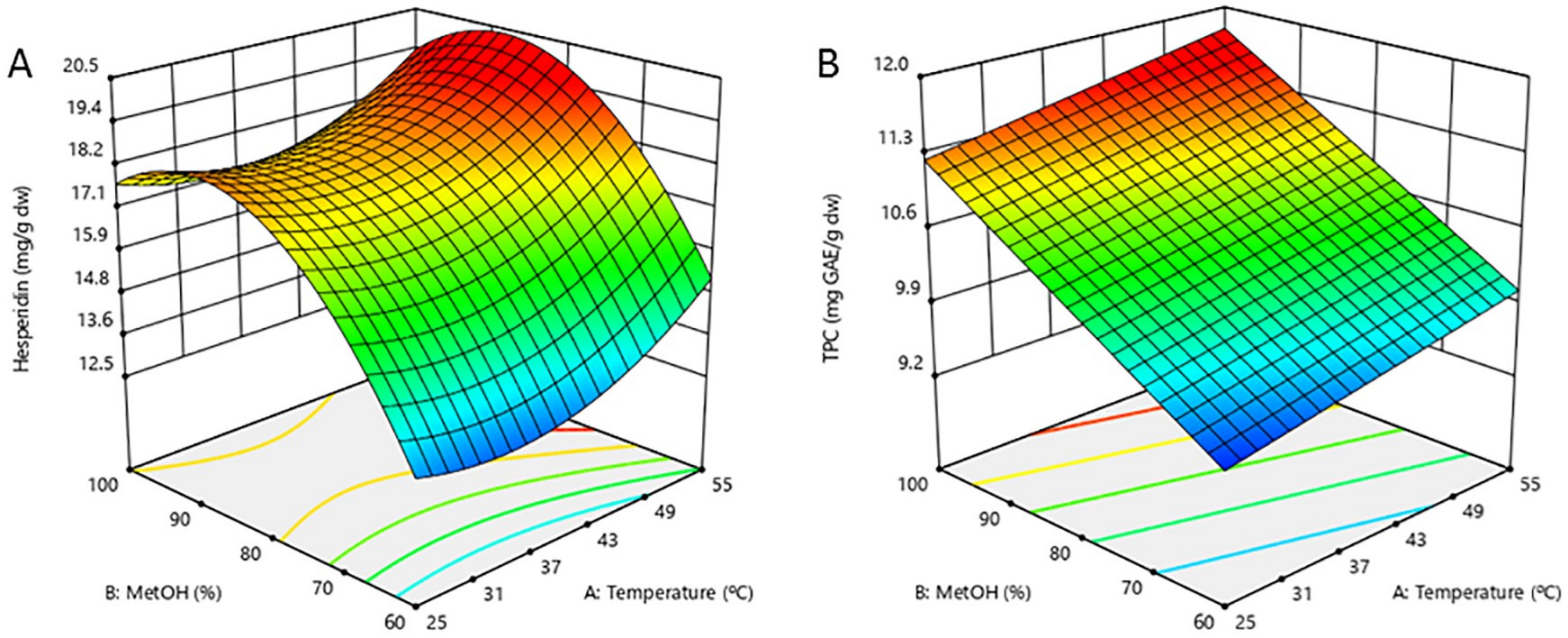
Response surface plots for hesperidin (A) and the total phenolic content (B) from *Citrus sinensis* pulp at a functional methanol proportion (MetOH) and temperature (T^a^).

The regression coefficients for the TPC and hesperidin models, reported in Table 2, allow for the interpretation of the effects of the independent variables on their extraction. Surface plots (Fig 2) facilitated the visualization of the significance of the extraction variables on the extraction of TPC and hesperidin.

Regarding the extraction temperature, the linear positive and negative effects were found to be statistically significant for TPC and hesperidin, respectively. For TPC, this indicates that increasing the temperature will result in greater TPC extraction (Fig 2B). Temperature usually produces positive linear and negative quadratic effects in RSM [9,13,17], including designs evaluating flavanones [19]. However, a significant positive quadratic effect was reported for hesperidin. This behavior has been previously reported for flavonols in *Inga edulis* leaves [9] and suggests that hesperidin requires more energetic conditions to be released into the solvent.

For the methanol proportion, positive linear effects were reported to be statistically significant for TPC and hesperidin. Additionally, a significant negative quadratic effect was found for hesperidin. This indicates that there is a maximum methanol proportion in the hesperidin extraction, after which its extraction starts to decrease; this effect can be observed in Fig 2A. Solvent positive linear and negative quadratic effects are widely reported in the literature [9,13]. In this sense, hesperidin and naringin extraction from citrus peels have been reported to present negative quadratic effects [19].

The combination of extraction variables at the highest desirability (0.971) was used to determine the optimal extraction conditions. In our study, the optimal extraction temperature was 55 °C. Similarly, orange peels have been reported to have an optimal extraction temperature of 60 °C for the extraction of total flavonoids, total phenolic compounds and antioxidant activity [18]. Additionally, TPC was reported to show an optimal extraction temperature at 55.56 °C in yuzu (*Citrus junos*) [19] and 48.3 °C in kaffir lime (*Citrus hystrix*) [17] peels. Further, 90% was found to be the optimal methanol proportion, possibly due to the low solubility of hesperidin in water [41]. Consistent with this result, high ethanol proportions have been reported as optimal for the extraction of phenolics from different citrus species [19,21]. For example, Silva *et al*. found 86.8% ethanol to be optimal for the extraction of *Inga edulis* phenolics at very similar conditions of 58.2 °C, 24 mL/g and 30 min [9]. Additionally, pure methanol is more efficient than methanol:water (50:50, v:v) for the extraction of lemon phenolics [38]. However, water ethanol mixtures of approximately 50% are optimal for the extraction of phenolics from bitter orange (*Citrus aurantium*) flowers [42] and kaffir lime (*Citrus hystrix*) peels [17].

#### Model validation

To validate the veracity of the model, 3 extractions were performed using the optimized conditions. No differences were obtained between the predicted and experimental values (Table 3). Therefore, the model accurately predicted the behavior of the response variables within the studied range for the extraction variables. Consequently, the extraction temperature and methanol proportion were fixed at 55 °C and 90%, respectively, throughout the rest of the study. Remarkably, the optimized solvent percentage significantly differed from other optimized methods for citrus peels [17,19]. This result demonstrates that each fruit matrix requires specific extraction conditions and studies such as ours for each specific fruit.

**Table 3:** Overall optimal values for the extraction parameters for hesperidin and the total phenolic content in *Citrus sinensis* pulp.

Model parameters			Response	Predicted value	Experimental value
T^a^ (°C)	MetOH (%)	Desirability			
55	90	0.971	Hesperidin	20.34	19.55 ± 0.52
			TPC	11.35	11.23 ± 0.29

Abbreviations: T^a^, Temperature; MetOH, methanol; and TPC, total polyphenol content. The results are expressed as mg of hesperidin or gallic acid equivalents per g of dry weight ± SD (n = 3).

### Effect of time on total polyphenol content

**Fig 3:**
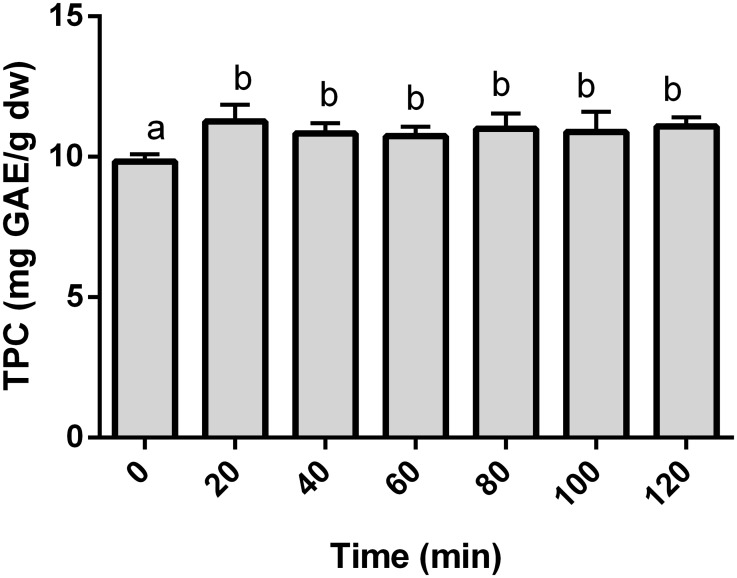
Effect of extraction time on the extraction of total phenolic compounds from *Citrus sinensis* pulp. The results (n = 3, each) are expressed as mg of gallic acid equivalents per g of dry weigh ± SD (mg GAE/g dw). Statistical analysis was performed by one-way ANOVA with Tukey’s post-hoc test (significant at p<0.05).

Theoretically, working at the optimal methanol proportion and temperature for a certain period of time allows the solubilization of phenolic compounds due to weakened cell wall interactions with phenolic compounds, leading to a greater transference of phenolics into the extraction solvent [11,14]. To test this in our orange sample, a range of extractions between 0 and 120 min was performed under the optimized conditions of 55 °C, 90% of methanol and 20 mL/g. Our results show that TPC increases at 20 min of extraction time, after which additional time does not produce any significant increases or decreases on the TPC (Fig 3). In this sense, increases in the extraction of different phenolic families due to time can reach a plateau after which they do not become more concentrated [9,39]. Importantly, the extraction of polyphenols from citrus peels requires extraction times approximately 120 min [17,19], further proving that the fruit matrix plays an important role in the optimal extraction conditions for citrus phenolics. Considering practical and economic issues, 20 min was fixed throughout the rest of the study.

### Effect of sequential steps on the total polyphenol content and hesperidin content

**Fig 4:**
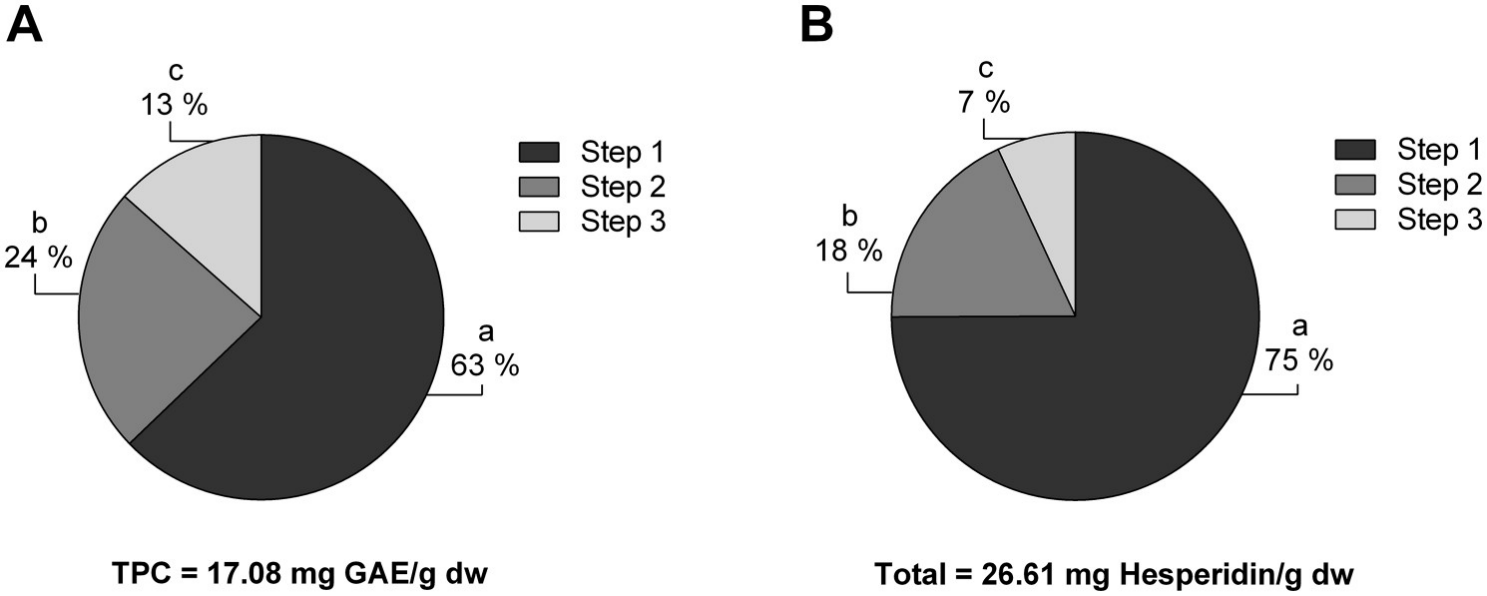
Effect of sequential extractions on the extraction of hesperidin (A) and the total phenolic compounds (B) from *Citrus sinensis* pulp. The results (n = 3, each) are expressed as mg of hesperidin or gallic acid equivalents per g of dry weigh ± SD (mg GAE or hesperidin/g dw).

To test whether our optimized method was able to extract most of the TPC and hesperidin from oranges in a single step, orange powder was subjected to three consecutive extractions under the optimized conditions (55 °C, 90%, 20 mL/g, and 20 min). Most of the hesperidin (93%) and TPC (87%) were extracted in the two first extraction steps (Fig 4). Because only 1.84 ± 0.12 mg hesperidin/g dw and 2.30 ± 0.34 mg GAE/g dw remained to be extracted in the third extraction, 2 sequential steps was selected as the optimized number of extractions. In agreement with this result, Mané *et al*. reported few increases in flavanol extraction with two, three and four consecutive extractions for grape skins, seeds and pulp [43]. Additionally, Yang *et al*. reported the same tendency for *Phyllanthus emblica L*. barks [11]. Remarkably, this method obtained 24.77 ± 1.28 mg hesperidin/g dw with two consecutive extractions.

### Investigation of methanol replacement for ethanol

**Fig 5:**
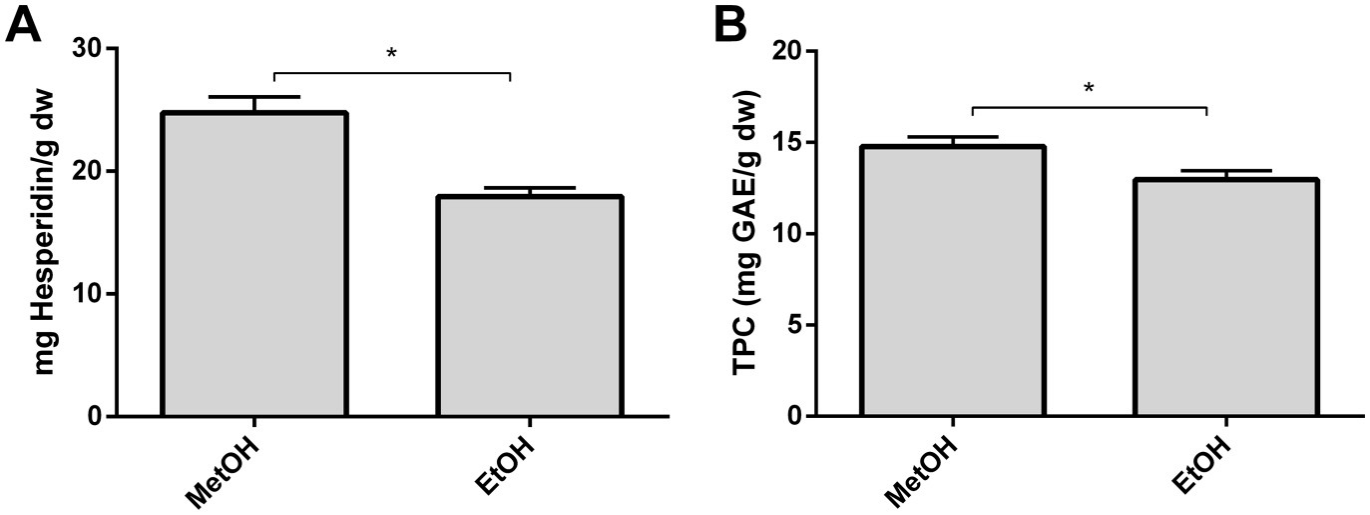
Comparison between the extraction efficiency of methanol (MetOH) and ethanol (EtOH) on the extraction of hesperidin (A) and the total phenolic compounds (B) from *Citrus sinensis* pulp. The results (n = 3, each) are expressed as mg of hesperidin or gallic acid equivalents per g of dry weigh ± SD (mg GAE or hesperidin/g dw). Statistical analysis was performed using Student’s t-test (significant at p<0.05).

Orange pulps are food industry by-products and could potentially be revalorized to obtain phenolic-rich extracts [29]. Ethanol is used in the food industry due to methanol’s toxicity [8]. To evaluate whether ethanol could replace methanol as the extraction solvent, NO powder was subjected to two consecutive extractions under the optimized conditions of 55 °C, 90% organic solvent, 20 mL/g, and 20 min agitation. Hesperidin and TPC were found to be statistically higher in methanolic extractions than in ethanolic extractions (Fig 5), in agreement with the fact that methanol usually achieves higher extraction rates than ethanol [8,15]. Indeed, Maguaza *et al*. reported a higher extraction of hesperidin from mandarin (*Citrus reticulata*) rinds when methanol was used as an extraction solvent [37]. Additionally, methanol has previously been used to extract phenolics from citrus species [10,17]. The fact that a fair amount of the total phenolics and hesperidin was extracted (17.93 ± 0.70 mg hesperidin/g dw and 12.95 ± 0.51 mg GAE/g dw) using ethanol as an extraction solvent and the important health activities of hesperidin [1–3] opens the door to possible adaptations of this method to produce phenolic-rich extracts with potential bioactive functions.

### Detailed characterization of sweet oranges

Once the extraction method was optimized (two sequential extractions at 55 °C, 90% MetOH, 20 mL/g and 20 min agitation), it was used to study the differences in the phenolic profiles generated by different geographical growing regions. To complete this objective, the phenolic profile of NO and SO pulps were compared. Characterization of the phenolic profiles of both orange cultivars was performed using an HPLC-ESI-MS/MS methodology. The phenolic profiles of the pulps from both oranges (Table 4) is in agreement with the phenolic profiles of juices from different sweet orange varieties [5,22,34]. In both studied sweet oranges, hesperidin dominated narirutin, which is in accordance with the phenolic profiles of orange juices [5]. Compounds such as didymin, kaempferol-3-*O*-rutinoside, protocatechuic acid *O*-glucoside, neoeriocitrin and rutin were found in lower but still relevant concentrations compared to hesperidin and narirutin. This trend has previously been found for different phenolics in several sweet orange varieties [22,34]. Minor phenolic compounds such as eriocitrin, *p*-coumaric acid, chlorogenic acid and ferulic acid were also found in orange pulp, in agreement with different studies in orange juices [5,6]. Previous studies have evaluated the phenolic profiles of orange pulps. However, only the total phenolic content and the content of flavanone aglycones after hydrolysis were evaluated [26–28]. Hence, this study provides an accurate and complete characterization of the phenolic contents found in sweet orange pulps.

**Table 4:** The phenolic contents from Navelina sweet orange pulps from the northern (NO) and southern (SO) hemispheres by HPLC-ESI-MS/MS expressed as mg/kg dw ± SD (n = 3, each).

Compound	NO	SO	p-value
**Benzoic Acid**	n.q.	n.d.	
**Phloroglucinol**	2.34 ± 0.13	n.d.	<0.01
**Hydorxybenzoic acid** ^a^	n.q.	n.q.	
**Protocatechuic acid**	3.89 ± 0.10	n.q.	
**p-Coumaric acid**	0.28 ± 0.00	0.09 ± 0.01	<0.01
**Gallic acid**	n.d.	n.q.	
**Caffeic acid**	n.q.	n.d.	
**Ferulic acid**	1.48 ± 0.02	0.84 ± 0.03	<0.01
**Apigenin**	n.q.	n.q.	
**Naringenin**	0.90 ± 0.08	0.25 ± 0.03	<0.01
**Kaempferol**	n.d.	n.d.	
**Eriodictyol**	n.q.	0.10 ± 0.00	
**Diosmetin**	n.q.	n.q.	
**Quercetin**	n.d.	n.d.	
**Hesperetin**	2.13 ± 0.12	1.85 ± 0.61	0.48
**Protocatechuic acid *O*-glucoside** ^b^	315.59 ± 20.95	254.88 ± 20.49	0.02
**p-Coumaric acid *O*-glucoside** ^c^	3.30 ± 0.09	2.22 ± 0.10	<0.01
**Gallic acid *O*-glucoside** ^d^	0.01 ± 0.00	0.01 ± 0.00	<0.01
**Caffeic acid *O*-glucoside d1** ^e^	10.25 ± 0.40	8.19 ± 0.56	0.01
**Caffeic acid *O*-glucoside d2** ^e^	23.73 ± 0.65	10.89 ± 0.42	<0.01
**Caffeic acid *O*-glucoside d3** ^e^	90.39 ± 3.18	36.14 ± 1.24	<0.01
**Chlorogenic acid d1** ^f^	2.64 ± 0.12	0.48 ± 0.22	<0.01
**Chlorogenic acid d2** ^f^	0.93 ± 0.02	n.q.	<0.01
**Chlorogenic acid**	7.70 ± 0.29	0.69 ± 0.03	<0.01
**Feruloylquinnic acid d1** ^g^	0.14 ± 0.00	0.13 ± 0.00	0.02
**Feruloylquinnic acid d2** ^g^	0.23 ± 0.01	n.d.	
**Phloridzin** ^h^	n.d.	0.45 ± 0.02	
**Kaempferol-3-*O*-glucoside**	n.q.	0.07 ± 0.01	
**Eriodictyol-7-*O*-glucoside**	n.d.	0.50 ± 0.03	
**Isorhamnetin-3-*O*-glucoside**	0.07 ± 0.00	0.06 ± 0.00	<0.01
**Myricetin-*O*-glucoside** ^i^	0.20 ± 0.00	0.11 ± 0.00	<0.01
**Narirutin** ^j^	8.07x10^3^ ± 0.62x10^3^	3.54x10^3^ ± 0.578x10^3^	<0.01
**Naringin**	n.d.	n.d.	
**Kaempferol-3-*O*-rutinoside**	516.20 ± 46.00	457.77 ± 16.52	0.11
**Didymin** ^k^	545.70 ± 52.62	444.57 ± 85.08	0.15
**Eriocitrin** ^l^	1.39 ± 0.07	0.73 ± 0.05	<0.01
**Neoeriocitrin** ^l^	127.13 ± 7.14	44.44 ± 6.49	<0.01
**Rutin**	91.23 ± 6.51	16.56 ± 0.79	<0.01
**Hesperidin**	38.28x10^3^ ± 3.91x10^3^	25.83x10^3^ ± 5.87x10^3^	0.06

d1, d2 and d3 indicate different isomeric compounds.

^a^ Quantified using the calibration curve of benzoic acid.

^b^ Quantified using the calibration curve of protocatechuic acid.

^c^ Quantified using the calibration curve of p-coumaric acid.

^d^ Quantified using the calibration curve of gallic acid.

^e^ Quantified using the calibration curve of caffeic acid.

^f^ Quantified using the calibration curve of chlorogenic acid.

^g^ Quantified using the calibration curve of ferulic acid.

^h^ Quantified using the calibration curve of phloroglucinol.

^i^ Quantified using the calibration curve of hyperoside.

^j^ Quantified using the calibration curve of naringin.

^k^ Quantified using the calibration curve of hesperidin.

^l^ Quantified using the calibration curve of eriodictyol-7-*O*-glucose.

Abbreviations: n.d., not detected; n.q., not quantified.

Statistical analysis was performed using Student’s t-test.

Phenolic compounds are plant-stress metabolites. Environmental conditions, which include temperature, water accessibility and light exposure, are known to modulate the phenolic profile of different fruits [44]. In this sense, SO, originally from the region of Mendoza (Argentina), were exposed to a temperate-arid climate and abundant solar exposure [45]. On the other hand, NO, originally from the region of Valencia (Spain), were exposed to a Mediterranean climate, characterized by mild and wet winters and hot and dry summers [46]. Therefore, changes in the phenolic profiles of oranges could be explained by their different geographical production regions. Indeed, important changes on amount and type of phenolic representatives have been linked to different growing regions on fruits with a wide representation of different phenolic families such as grapes [47], pomegranates [48] and tomatoes [49]. Importantly, many of the factors that can modulate the phenolic compounds in fruits are triggered by changes in the expression of relevant polyphenol biosynthetic genes [50]. However, in terms of the phenolic representatives, NO and SO were very similar, though the amounts of phenolic compounds reported in each cultivar were different. In general, most phenolic compounds reported significantly higher concentrations in NO than in SO. Considering that oranges mainly express the genes involved in the flavanone biosynthetic pathway [1,51], the differences in growing conditions conditioned by SO and NO production region must have led to the activation or inhibition of the expression of flavanone biosynthetic genes, thus involving that the only difference between SO and NO is the total amount of phenolic compounds and not the type of representatives. In agreement with our results, differences in the content of individual phenolic compouns were found in other citrus species grown such as pummelo (*Citrus grandis*) [52], bergamot (*Citrus bergamia*) [53] and lemons (*Citrus limon*) [54] grown in different regions. Surprisingly, no significant differences were reported for the content of the major phenolic, hesperidin, in sweet oranges (*p* = 0.06). Our results indicate that the region of cultivation has an impact on the concentrations of individual phenolic compounds found in sweet orange pulps.

## Conclusions

In this study, we developed an optimized extraction method that is capable of extracting *Citrus sinensis* phenolics from its pulp. This method consists of two relatively fast sequential extractions (20 min each) using a relatively mild temperature (55 °C) and a high methanol concentration (90%). Importantly, our method is able to extract high quantities of phenolic compounds, which is essential for determining the accurate phenolic profile present in the fruit. Given that no previous extraction methods for orange pulps are reported in the literature, we propose this method as a routine methodology to study the phenolic profile of the edible parts of sweet oranges. Additionally, this method could be useful for producing phenolic-rich extracts with potential bioactive functions. This study also indicates that the geographical region affects the phenolic profile of orange pulps.

## Supporting information

S1 Table HPLC-DAD method parameters for hesperidin. ^a^ μg/mL. Abbreviations: R^2^, determination coefficient; LOD, limit of detection; and LOQ, limit of quantification.
(PDF)

S2 Table Molecular weight, detected ion mass by qTOF and optimized MRM (multiple reaction monitoring) conditions for the identified polyphenol compounds in Navelina sweet orange pulps by HPLC-ESI-MS/MS. Abbreviations: MW, molecular weight; CE, collision energy; Ms>Ms transitions.
(PDF)

S3 Table HPLC-ESI-MS/MS method quality parameters for the study of polyphenol compounds in Navelina sweet orange pulps. (PDF)

S1 Fig Total Ion Chromatogram (TIC) of a NO sample analyzed by HPLC-ESI-MS/MS in this study. (TIF)

S2 Fig HPLC-ESI-MS/MS chromatograms of all the quantified compounds in SO and NO in this study. (TIF)

S3 Fig Spectra of all quantified compounds in SO and NO in this study. (TIF)

## Abbreviations

GAE: gallic acid equivalents
LSR: liquid-t-solid ratio
MetOH: methanol
RSM: response surface methodology
TPC: total polyphenol content
