# Supplementary material for: Optimization of a polyphenol extraction method for sweet orange pulp (Citrus sinensis L.) to identify phenolic compounds consumed from sweet oranges
Source: PLoS One. 2019 Jan 30;14(1):e0211267. doi: 10.1371/journal.pone.0211267 (PMC6353169; doi:10.1371/journal.pone.0211267)
Supplement: S1 Table — a μg/mL. Abbreviations: R2, determination coefficient; LOD, limit of detection; and LOQ, limit of quantification. (PDF) [file pone.0211267.s001.pdf]

Supplementary Table 1: HPLC-DAD method parameters for hesperidin.

Compound	Detection wavelength (nm)	Calibration curve	R^2	Linearity[a]	Precision (% RSD, n=3)[b]			Reproducibility (% RSD, n=3)[b]			LOD[a]	LOQ[a]
					1	20	50	1	20	50		
Hesperidin	280	Y= 8.24X	>0.99	3.31-165.41	0.70	0.78	1.96	2.44	0.53	2.06	0.42	1.40

[a] µM. [b] µg/mL.

Abbreviations: R^2, determination coefficient; LOD, limit of detection; and LOQ, limit of quantification.
