# Supplementary material for: Optimization of a polyphenol extraction method for sweet orange pulp (Citrus sinensis L.) to identify phenolic compounds consumed from sweet oranges
Source: PLoS One. 2019 Jan 30;14(1):e0211267. doi: 10.1371/journal.pone.0211267 (PMC6353169; doi:10.1371/journal.pone.0211267)
Supplement: S2 Table — Abbreviations: MW, molecular weight; CE, collision energy; Ms>Ms transitions. (PDF) [file pone.0211267.s002.pdf]

Supplementary Table 2: Molecular weight, detected ion mass by qTOF and optimized MRM (multiple reaction monitoring) conditions for the identified polyphenol compounds in Navelina sweet orange pulps by HPLC-ESI-MS/MS.

Compound	MW	$[M-H]^-$	Quantification		Confirmation	
			MS/MS	CE (V)	MS/MS	CE (V)
**Benzoic acid**	122.12	121.0304	121>77	8	121>59	4
**Phloroglucinol**	126.11	125.0265	125>57	20	125>125	0
**Hydroxybenzoic acid**	138.12	137.0256	137>93	40	137>119	40
**Protocatechuic acid**	154.12	153.0215	153>109	16	153>62	40
**p-Coumaric acid**	164.16	163.0774	163>119	16	163>93	36
**Gallic Acid**	170.12	169.0137	169>125	12	169>79	24
**Caffeic acid**	180.16	179.0344	179>135	16	179>107	24
**Ferulic acid**	194.18	193.052	193>134	12	193>178	12
**Apigenin**	270.24	269.0452	269>117	44	269>151	28
**Naringenin**	272.25	271.0619	271>151	20	271>119	32
**Kaempferol**	286.24	285.0403	285>239	28	285>117	56
**Eriodictyol**	288.25	287.0793	287>151	12	271>135	28
**Disometin**	300.26	299.0565	299>284	20	299>256	36
**Quercetin**	302.24	301.0100	301>151	20	301>179	20
**Hesperetin**	302.28	301.0525	301>164	28	301>286	16
**Protocatechuic acid glucoside**	316.26	315.0737	315>153	10	315>109	20
**hGallic acid O-glucoside**	332.26	341.0896	331>169	12	331>125	12
**Caffeic acid O-glucoside**	342.30	341.0896	341>179	20	341>323	10
**Chlorogenic acid**	354.31	353.0905	353>191	16	353>85	16
**Feruloylquinnic acid**	368.34	367.1062	367>193	40	367>161	40
**Phloridzin**	436.41	435.1331	435>273	10	435>167	40
**Kaempferol-3-*O*-glucoside**	448.38	447.0974	447>284	28	447>255	40
**Eriodictyol-7-*O*-glucoside**	450.39	449.1084	449>287	12	449>151	36
**Hyperoside**	464.38	463.0917	463>300	32	463>271	48
**Isorhamnetin-3-*O*-glucoside**	478.40	477.1014	477>314	32	477>285	40
**Myricetin-3-*O*-glucoside**	480.38	479.0841	479>317	20	479>461	10
**Narirutin**	580.53	579.1762	579>271	40	579>151	48
**Naringin**	580.53	579.1762	579>271	40	579>151	48
**Kaempferol-3-*O*-rutinoside**	594.52	593.1560	593>353	32	593>255	60
**Didymin**	594.56	593.1963	593>285	20		
**Eriocitrin**	596.53	595.1705	595>287	20		
**Neoeriocitrin**	596.53	595.1711	595>287	20		
**Rutin**	610.52	609.1508	609>300	40	609>271	60
**Hesperidin**	610.56	609.1869	609>301	20	609>286	52

Abbreviations: MW, molecular weight; CE, collision energy; Ms>Ms transitions.
