# Supplementary material for: Optimization of a polyphenol extraction method for sweet orange pulp (Citrus sinensis L.) to identify phenolic compounds consumed from sweet oranges
Source: PLoS One. 2019 Jan 30;14(1):e0211267. doi: 10.1371/journal.pone.0211267 (PMC6353169; doi:10.1371/journal.pone.0211267)
Supplement: S3 Table — (PDF) [file pone.0211267.s003.pdf]

Supplementary Table 3: HPLC-ESI-MS/MS method quality parameters for the study of polyphenol compounds in Navelina sweet orange pulps.

Compound	RT (min)	Calibration curve	R^2	Linearity (μM)	LOD (nM)	LOQ (nM)
**Benzoic acid**	9.77	y=304.74x	0.993	0.016-40.943	7.18	23.94
**Phloroglucinol**	2.80	y=203.80x	0.992	0.016-39.648	0.40	1.33
**Protocatechuic acid**	3.90	y=11.11x	0.996	0.013-32.442	18.20	60.68
**p-Coumaric acid**	7.68	y=3567.30x	0.999	0.012-30.458	0.87	2.89
**Gallic Acid**	3.02	y=11453.00x	0.996	0.012-29.391	0.10	0.33
**Caffeic acid**	5.85	y=88.55x	0.999	0.011-27.754	4.73	15.76
**Ferulic acid**	8.32	y=1019.40x	0.996	0.010-25.749	0.43	1.43
**Apigenin**	14.57	y=1950.50x	0.992	0.007-18.502	0.10	0.32
**Naringenin**	14.62	y=864.67x	0.999	0.007-18.365	0.18	0.61
**Kaempferol**	13.03	y=1489.30x	0.997	0.007-17.468	0.16	0.49
**Eriodictyol**	12.84	y=1556.40x	0.995	0.007-17.346	0.05	0.15
**Disometin**	14.81	y=2782.00x	0.997	0.007-16.543	0.14	0.47
**Quercetin**	13.25	y=1628.00x	0.996	0.007-16.543	0.05	0.17
**Hesperetin**	14.99	y=520.95x	0.995	0.007-16.541	0.08	0.25
**Chlorogenic acid**	4.80	y=1032.10x	0.999	0.006-14.112	0.32	1.07
**Kaempferol-3-*O*-glucoside**	9.55	y=1073.20x	0.993	0.004-11.151	0.06	0.19
**Eriodictyol-7-*O*-glucoside**	8.44	y=1968.60x	0.995	0.004-11.101	0.04	0.15
**Hyperoside**	8.37	y=1527.80x	0.994	0.004-10.767	0.04	0.14
**Isorhamnetin-3-*O*-glucoside**	9.71	y=1058.90x	0.990	0.004-10.451	0.04	0.15
**Naringin**	9.57	y=591.45x	0.997	0.003-8.613	0.14	0.48
**Kaempferol-3-*O*-rutinoside**	9.04	y=976.41x	0.998	0.003-8.410	0.03	0.09
**Rutin**	8.07	y=825.00x	0.998	0.003-8.190	0.06	0.20
**Hesperidin**	9.02	y=938.04x	0.994	0.003-8.189	0.52	1.74
