# Supplementary figures and images for: Optimization of a polyphenol extraction method for sweet orange pulp (Citrus sinensis L.) to identify phenolic compounds consumed from sweet oranges
Source: PLoS One. 2019 Jan 30;14(1):e0211267. doi: 10.1371/journal.pone.0211267 (PMC6353169; doi:10.1371/journal.pone.0211267)

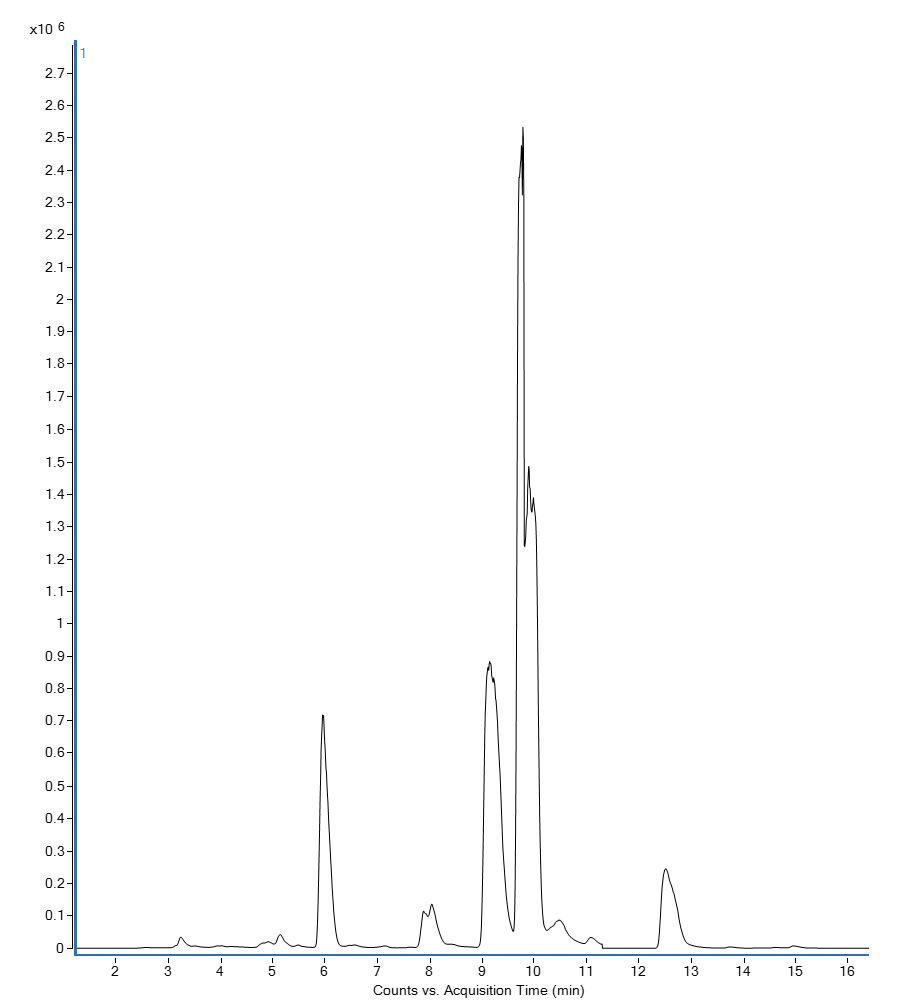

Supplement: S1 Fig — (TIF) [file pone.0211267.s004.tif]

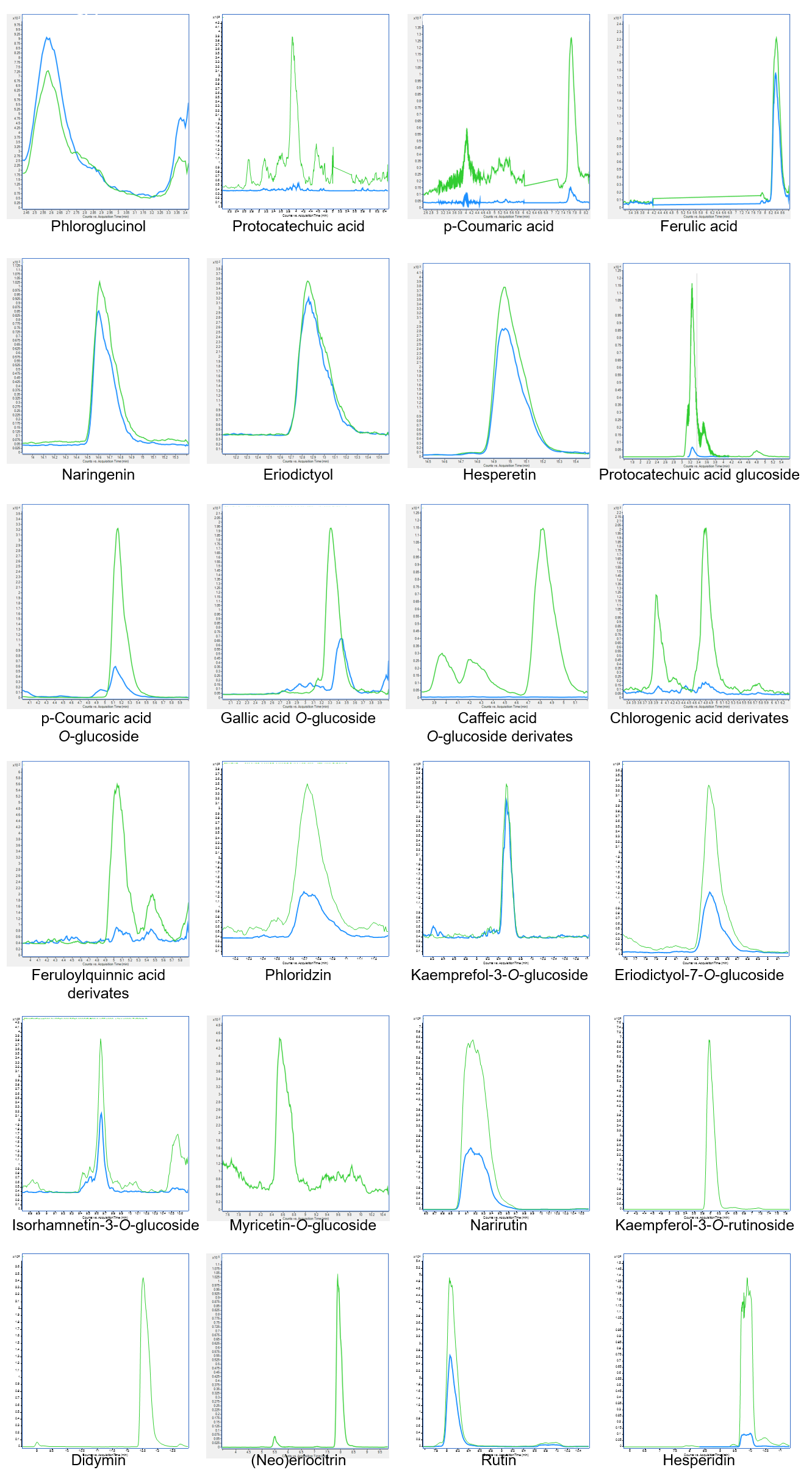

Supplement: S2 Fig — (TIF) [file pone.0211267.s005.tif]

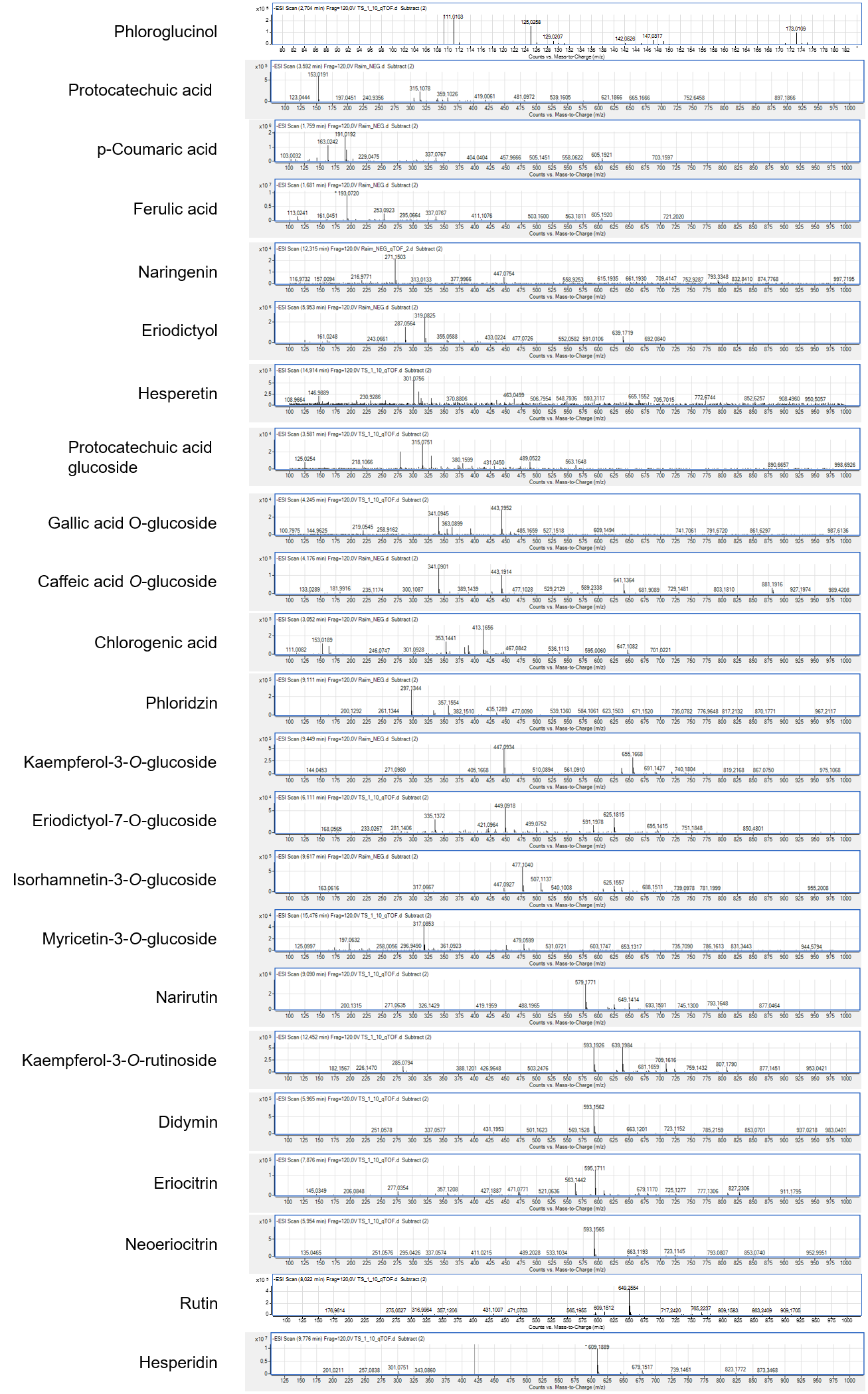

Supplement: S3 Fig — (TIF) [file pone.0211267.s006.tif]
